# Retinoic acid metabolism related gene CYP26B1 promotes tumor stemness and tumor microenvironment remodeling in bladder cancer

**DOI:** 10.7150/jca.101406

**Published:** 2025-04-22

**Authors:** Jun Gu, Zhen-Duo Shi, Kun Pang, Lin Hao, Wei Wang, Cong-hui Han

**Affiliations:** 1 Suzhou Medical College of Soochow University, Suzhou, China.; 2 Department of Urology, Xuzhou Central Hospital, (Clinical Medicine Postgraduate Workstation, Soochow University), Xuzhou 221009, China.; 3 The Affiliated Jiangsu Shengze Hospital of Nanjing Medical University, China.; 4 Department of Medical College, Southeast University, 87 DingjiaQiao, China.

## Abstract

**Background**: The previous studies have shown that the retinoic acid (RA) metabolism is closely related to the cancer stemness, but its role in bladder cancer development has not been fully investigated.

**Methods**: We conducted a comprehensive analysis of mutations, copy number variations and transcriptional changes of RA metabolism related genes in bladder cancer cells. We evaluated the activity of RA metabolism in tumor cells by using single cell transcriptome data and identified differentially expressed genes (DEGs) in cell subsets with high RA-metabolism score. We also investigated and verified the biological function of *CYP26B1* (one of RA metabolism related genes) *in vitro*. Additionally, we analyzed and verified the relationship between *CYP26B1* and tumor immune microenvironment by multiplex immunohistochemical (mIHC).

**Results**: Comprehensive analysis indicates that the mutation rate of RA metabolism related genes in bladder cancer is about 20%, with significant gene amplification observed in* RDH10* and *CYP26B1.* We identified a group of subsets with significantly increased RA metabolism activity in bladder cancer tumor epithelial cells and found that this subgroup was significantly associated with poor prognosis (p < 0.05). *CYP26B1* was identified as a potential therapeutic target. It was found that *CYP26B1* is significantly correlated with tumor stemness and differentiation. *In vitro* experiments confirmed that overexpression of *CYP26B1* can significantly enhance the proliferation and migration of tumor cells.

**Conclusion:** These results suggest that CYP26B1 may be closely related to the remodeling of the tumor microenvironment and may become a potential therapeutic target for bladder cancer.

## Introduction

Bladder cancer (BLCA) is one of the most prevalent cancers worldwide and the second-largest malignancy of the urinary system, accounting for approximately 10% of all urinary system tumors 1, 2. It is a disease strongly associated with risk factors such as smoking, chemical exposure, and chronic cystitis, with smoking being the most significant 3, 4. Early diagnosis plays a pivotal role in improving treatment outcomes for BLCA patients. Advances in medical imaging techniques, such as cystoscopy and CT scans, have revolutionized the ability to detect tumors early and assess their location and size, thereby enabling better treatment planning 5, 6. Additionally, non-invasive bladder tumor markers identified through urine tests have shown promise in detecting BLCA at an earlier stage, which is crucial for improving prognosis.

Current treatment modalities for bladder cancer include surgical resection, radiotherapy, and chemotherapy. Early-stage tumors are typically managed through surgery, which offers the best outcomes, while advanced stages often require adjuvant therapies such as chemotherapy and radiotherapy. In recent years, immunotherapy and targeted therapies have emerged as new focal points of research, showing great potential in enhancing therapeutic outcomes and minimizing side effects. However, despite these advancements, the prognosis for patients with advanced or metastatic BLCA remains poor, highlighting the need for the development of novel therapeutic strategies. As the incidence of bladder cancer continues to rise globally, early detection, comprehensive treatment strategies, and innovative therapies are paramount to improving patient outcomes.

One area of significant interest in cancer research is the role of retinoic acid (RA) metabolism, which is intricately involved in regulating various cellular processes, including differentiation, proliferation, and apoptosis^7^, ^8^. RA is a bioactive form of vitamin A and regulates gene expression by activating its nuclear receptors (RARs), which come in three subtypes: RARα, RARβ, and RARγ 9 10. This regulation plays a crucial role in maintaining normal cell development and growth. Dysregulation of RA metabolism has been implicated in the pathogenesis of several cancers, as it can lead to altered cellular differentiation and proliferation, processes that are commonly disrupted in cancerous tissues.

Interestingly, cancer can be viewed as a form of developmental disorder, in which the regulatory mechanisms governing cell growth and differentiation go awry. Retinoic acid's potential as an anti-cancer agent has been well-documented, particularly in the treatment of acute promyelocytic leukemia (APL), where RA is used to induce differentiation and remission 11. For example, the RARβ2 splicing variant is a well-known tumor suppressor that is silenced epigenetically in human cancers^12^. Furthermore, RA has been shown to impact cancer stem cells (CSCs), which are thought to arise from tissue-specific stem or progenitor cells and contribute to tumor initiation, progression, and relapse. While some CSCs are quiescent and thus resistant to traditional chemotherapy, RA may have the ability to modulate these cells, offering a potential strategy for targeting therapy-resistant populations 13 14. Although the role of RA in cancer stem cells has been well studied in other malignancies, its function in bladder cancer remains underexplored.

The relationship between RA metabolism and the tumor immune microenvironment has also garnered significant attention in recent years. RA plays a critical role in regulating immune responses, including the activity of T cells, macrophages, and natural killer cells, all of which are central to the body's ability to recognize and eliminate tumor cells 15 16 17. RA influences both the tumor cells and the immune microenvironment, shaping tumor progression and metastasis. Notably, mutations or abnormal expression of genes involved in RA metabolism—such as the tumor suppressor gene p53—can lead to reduced RA levels and impaired immune responses, thereby promoting tumor growth and immune evasion 18. Therefore, understanding the intricate relationship between RA metabolism and the tumor immune microenvironment could open the door to innovative therapeutic strategies that target both tumor cells and the immune response.

Among the key enzymes involved in RA metabolism, CYP26B1, a member of the cytochrome P450 family, plays a pivotal role in the degradation of RA^19^. CYP26B1 has been linked to various cancers due to its ability to modulate RA levels^20^. However, its role in bladder cancer (BLCA) had not been well defined prior to this study. CYP26B1's function in degrading RA suggests that its dysregulation could lead to altered RA signaling, influencing both cancer cell behavior and the immune microenvironment. The expression and potential therapeutic targeting of CYP26B1 in bladder cancer could therefore offer a new avenue for treatment, similar to its role in other malignancies. Recent studies have highlighted CYP26B1 as a potential therapeutic target in cancers such as prostate and liver cancer, but its significance in bladder cancer has remained underexplored.

This study aims to fill this gap by examining the impact of CYP26B1 on bladder cancer progression and its association with the immune microenvironment. Using a combination of bioinformatics analysis and experimental validation, we explore how CYP26B1 influences both tumor growth and the immune landscape within bladder cancer. Our findings suggest that CYP26B1 could serve as a novel therapeutic target in BLCA, offering a potential strategy for modulating RA signaling and improving clinical outcomes. In summary, this study not only expands our understanding of the molecular mechanisms underlying bladder cancer but also emphasizes the therapeutic potential of targeting CYP26B1 in RA metabolism. As we continue to explore the complex interactions between RA, the immune microenvironment, and cancer stemness, we hope to contribute to the development of more effective, targeted therapies for bladder cancer.

## Materials and methods

### Data collection and normalization

We collected single-cell RNA sequencing (scRNA-seq) data from 10 bladder cancer samples across two publicly available datasets. Specifically, we utilized GSE130001 (comprising 2 samples) and GSE135337 (comprising 8 samples), both of which are accessible via the Gene Expression Omnibus (GEO) database (https://www.ncbi.nlm.nih.gov/geo/). Additionally, we retrieved bulk gene expression data and corresponding clinical data from 403 bladder cancer samples in the Cancer Genome Atlas (TCGA) cohort. An additional 165 bladder cancer samples were obtained from the GSE13507 dataset via GEO. Furthermore, we acquired the IMvigor210 cohort, a dataset containing information on individuals diagnosed with locally advanced or metastatic uroepithelial cancer who were treated with atezolizumab, through the following resource: https://research-pub.gene.com/IMvigor210CoreBiologies. Samples were excluded if their follow-up duration was shorter than 30 days. Sample details are summarized in Table 1.

### Exploring the RA metabolism landscape in bladder cancer

#### Identification of RA metabolism-related genes

To understand the role of retinoic acid (RA) metabolism in bladder cancer, we focused on 10 key RA metabolism-related genes: *LRAT*,* CES1*, *DHRS3*,* RDH10*,* ALDH1A1*,* ALDH1A2*,* ALDH1A3*,* CYP26A1*, *CYP26B1*, *CYP26C1^21^*.

#### Genetic alterations analysis

We first examined the mutational landscape and copy number variations (CNVs) of these RA metabolism-related genes in the bladder cancer cohort from TCGA using the CBioPortal tool 22. This allowed us to identify the frequency and types of alterations affecting these genes in bladder cancer samples.

#### DNA methylation analysis

Next, we explored the DNA methylation patterns of these RA metabolism-related genes. We performed a comparison of the DNA methylation levels between cancerous and adjacent normal tissues, assessing the epigenetic alterations that may contribute to cancer progression. We further analyzed the correlation between DNA methylation levels and the corresponding gene expression levels to investigate potential regulatory mechanisms.

#### Correlation with tumor signaling pathways

To gain deeper insights into the functional relevance of RA metabolism in bladder cancer, we assessed the correlation between the expression of these genes and key tumor-related signaling pathways, such as the cell cycle and DNA damage response. Additionally, we used Gene Set Variation Analysis (GSVA) to evaluate the correlation between RA metabolism scores and various tumor-related signaling pathways.

### Data Processing for scRNA-seq and Cell Type Identification

#### Quality Control and Preprocessing

We processed the raw scRNA-seq data using the Seurat package 23. Initially, Seurat objects were created with filtering parameters of min.cells = 3 and min.features = 200 to ensure high-quality data. We further filtered cells based on the number of detected genes per cell, excluding cells with fewer than 200 or more than 5000 genes. Cells with mitochondrial UMI counts greater than 10% were also excluded to avoid potential contamination from dying or stressed cells.

#### Variable gene selection and scaling

The expression data were scaled by identifying the top 2000 most variable genes using the FindVariableFeatures function. This allowed us to focus on the most biologically relevant genes in the context of bladder cancer. Subsequently, the scaled data were subjected to Principal Component Analysis (PCA) to reduce dimensionality and uncover key patterns in the data. PCA is used to reduce this high-dimensional data into a lower-dimensional space while retaining as much variance as possible.

#### Cell type identification and clustering

We performed data integration and clustering, which resulted in the identification of 21 distinct cell type clusters. To assign cell type identities, we utilized classical cell-specific markers, including: Epithelial cells: *KRT8*, *KRT18*, *EPCAM*; Myeloid cells: *CD14*, *AIF1*, *CD68*, *CD83*, *CSF1R*, *FCER1G*; Fibroblasts: *COL1A1*, *COL1A2*, *COL6A1*, *COL6A2*, *DCN*, *PDPN*, *TAGLN*; Endothelial cells: *VWF*, *PECAM1*, *CLND5*, *PLVAP*, *CDH5*; T cells: *CD3E*, *CD3G*, *CD3D*, *CD2*, *CD52*. We used ClusterGVis (https://github.com/junjunlab/ClusterGVis) for functional enrichment analysis of epithelial cells in each identified subcluster. Additionally, we estimated the RA metabolism score for each cell type using Seurat's AddModuleScore function.

### Exploring potential RA metabolism-related therapeutic targets

#### Protein-protein interaction network construction

To explore the functional network of RA metabolism-related genes, we constructed a protein-protein interaction (PPI) network using the GeneMANIA tool 24. This network helped us identify potential gene interactions and pathways associated with RA metabolism in bladder cancer.

#### RA metabolic score and prognosis

We collected the RA metabolism related gene sets and thus estimate the RA metabolism level in scRNA data by using AddMouduleScore function based on the expression level of the RA metabolism related genes. We calculated the RA metabolic score for each sample in the TCGA cohort using GSVA. The relationship between RA metabolic score and patient prognosis was analyzed by performing survival analysis, including Cox proportional hazards modeling and log-rank tests.

#### Differential gene expression analysis

To identify differentially expressed genes (DEGs) between high and low RA metabolic score groups, we used the LIMMA package 25. Genes with a log fold-change (logFC) ≥ 1 and a p-value < 0.05 were considered significant DEGs. These DEGs were further analyzed for their potential involvement in bladder cancer progression.

#### Target identification and validation

We identified overlapping DEGs between the TCGA bladder cancer cohort and single-cell data. From this list, two genes were selected as potential drug targets. We used Metascape 26 to perform pathway enrichment and functional analysis to predict the biological roles of these genes.

### Exploring the biological function of CYP26B1 in bladder cancer

#### Role of CYP26B1 in bladder cancer

Based on the scRNA-seq and TCGA data, we identified CYP26B1 as a critical RA metabolism-related gene in bladder cancer. Using the CancerSEA tool 27, we examined the relationship between CYP26B1 expression and key cancer-associated processes, including angiogenesis, epithelial-mesenchymal transition (EMT), and DNA damage repair.

#### Prognostic value of CYP26B1

We assessed the prognostic value of CYP26B1 by performing survival analysis using the Kaplan-Meier method. We also explored the potential association between CYP26B1 expression and DNA damage repair pathways.

#### *In vitro* functional assays

To investigate the functional role of CYP26B1 in bladder cancer, we performed transwell, wound healing, CCK-8, and clonogenic assays to evaluate the effects of CYP26B1 on cell migration, proliferation, and colony formation. 5637 and T24 bladder cancer cell lines were transfected with an empty vector or a CYP26B1-overexpression plasmid using Lipofectamine 3000 reagent (Invitrogen), following the manufacturer's instructions. Additionally, we treated cells with liarozole dihydrochloride (L-D) (Selleck Chemicals), a selective inhibitor of P450 enzymes, to inhibit CYP26B1 expression.

#### *In vivo* experiments

To investigate the impact of CYP26B1 on bladder cancer growth, we utilized a subcutaneous xenograft mouse model. Briefly, 5×10⁶ human bladder cancer 5637 cells were suspended in 100 µL of Matrigel and subcutaneously injected into the flank of 6-week-old female nude mice (BALB/c nude). Tumor growth was monitored by caliper measurements once weekly, and tumor volume was calculated using the formula: V = (L × W²)*0.52, where L is the length and W is the width of the tumor. Mice were randomly assigned to three experimental groups: (1) control, (2) CYP26B1 overexpression, and (3) CYP26B1 inhibition. For CYP26B1 overexpression, 5637 cells were transfected with a CYP26B1 overexpression plasmid prior to injection. For the CYP26B1 inhibition group, mice were treated with the CYP26B1 inhibitor Liarozole dihydrochloride (40 mg/kg, orally) starting from day 7 post-injection, and this treatment was continued daily until the end of the study. Tumor size and animal body weight were recorded weekly. After 4 weeks of treatment, mice were euthanized, and tumors were excised for further analysis.

### Exploring the associations between CYP26B1 and the tumor microenvironment

#### Correlation with tumor-infiltrating immune cells

Given the role of CYP26B1 in DNA damage repair, we explored its potential impact on the tumor immune microenvironment. Using the TISIDB database 28, we assessed the correlation between CYP26B1 expression and tumor-infiltrating immune cells.

#### Immunotherapy response

We evaluated the association between CYP26B1 expression and the response to atezolizumab (anti-PD-L1 therapy) in the IMvigor210 cohort.

#### Multiplex immunohistochemistry (mIHC)

To validate the expression of CYP26B1 in the tumor microenvironment, we performed multiplex immunohistochemistry (mIHC) on bladder cancer tissue samples. The following antibodies were used: anti-human CD3 (Abcam, ab135372), CD4 (Abcam, ab133616), CD8 (Abcam, ab217344), and CYP26B1 (Abcam, ab113640). Expression levels were quantified using Visiopharm software.

### Statistical analysis

Statistical analyses were performed using R and associated packages. Differential expression of genes was determined by calculating the fold change between tumor and normal tissue. A t-test was used to calculate p-values, with false discovery rate (FDR) correction applied. Survival analysis was conducted using the Cox proportional hazards model and Kaplan-Meier method, with a significance threshold of p-value < 0.05.

## Results

### The landscape of RA metabolism related genes in BLCA

**Figure 1:**
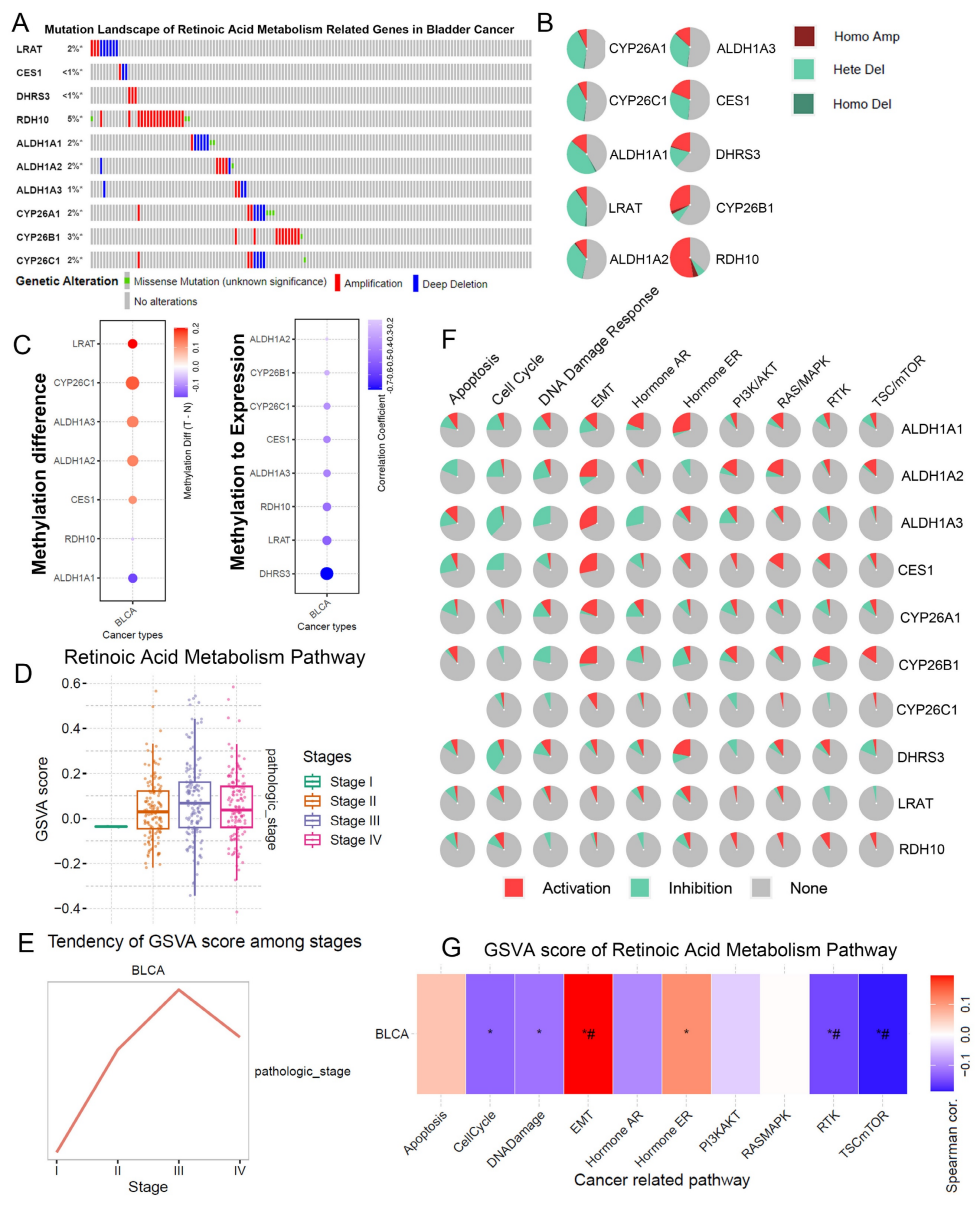
**The landscape of RA metabolism related genes in BLCA.** (A) Gene mutation data of RA metabolism related genes. (B) Copy number variation of RA metabolism related genes. (C) DNA methylation of RA metabolism related genes. (D-E) Associations between GSVA score (RA metabolism related genes) and clinical stage. (F) Function prediction of RA metabolism related genes. (G) Associations between GSVA score (RA metabolism related genes) and carcinogenic pathways.

We examined the mutation and copy number variations (CNVs) of RA metabolism-related genes in BLCA. The mutation rate of these genes in BLCA was observed to be nearly 20%, with significant gene amplifications in RDH10 and CYP26B1 (**Figure 1A, B**). This suggests that genomic alterations in RA metabolism pathways could play a role in the initiation and progression of BLCA. Notably, these findings align with prior studies showing that RA metabolism is implicated in various cancer types through its role in cell differentiation and growth regulation.

Furthermore, significant DNA methylation changes were observed in RA metabolism-related genes, particularly LRAT and CYP26C1, which showed hypermethylation in BLCA tumors compared to adjacent normal tissues (**Figure 1C**). The negative correlation between the methylation status of DHRS3, LRAT, and RDH10 and their gene expression levels suggests that methylation changes could regulate the expression of key genes involved in RA metabolism, possibly altering tumor behavior. These results support previous research indicating that epigenetic modifications play a critical role in tumorigenesis and the regulation of metabolic pathways.

In terms of tumor stage, a higher RA metabolism score was significantly correlated with more advanced stages of BLCA (**Figure 1D, E**). We found that RA metabolism-related genes were strongly associated with EMT and negatively correlated with the cell cycle, DNA damage, and repair. (**Figure 1F, G**). These findings support the idea that RA metabolism could facilitate BLCA progression by enhancing the metastatic potential of cancer cells, as observed in other malignancies.

### Identification of a RA metabolism related blca cancer subcluster from scRNA-seq data

**Figure 2:**
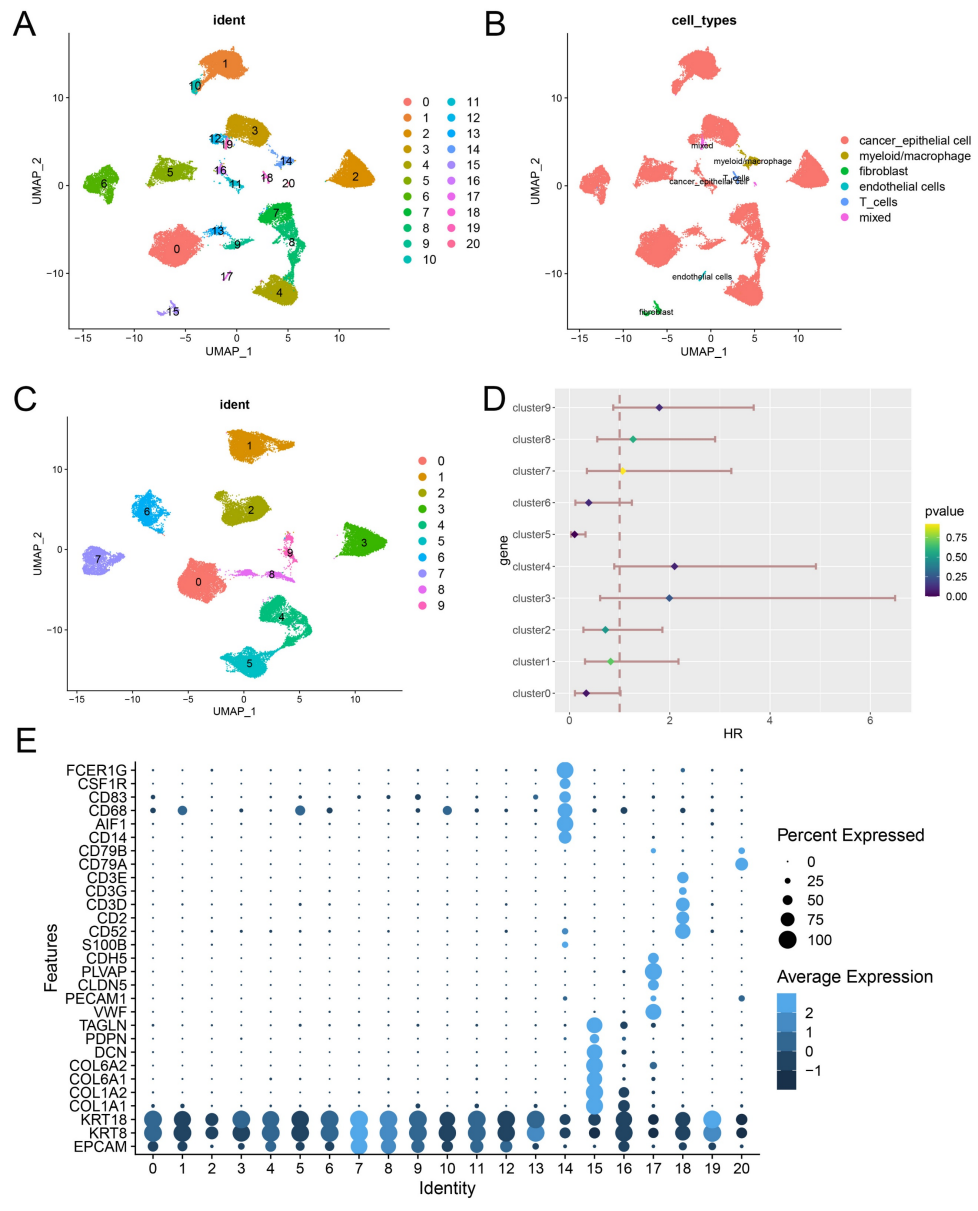
** Identification of a RA metabolism related blca cancer subcluster from scRNA-seq data.** (A) 21 potential cell subsets identified from single cell data of bladder cancer. (B) Cell type identification. (C) Re-grouping of epithelial cells of bladder tumor. (D) Potential prognostic implications of epithelial cell subsets in bladder tumors. (E) Display of classic cell typing markers.

**Figure 3:**
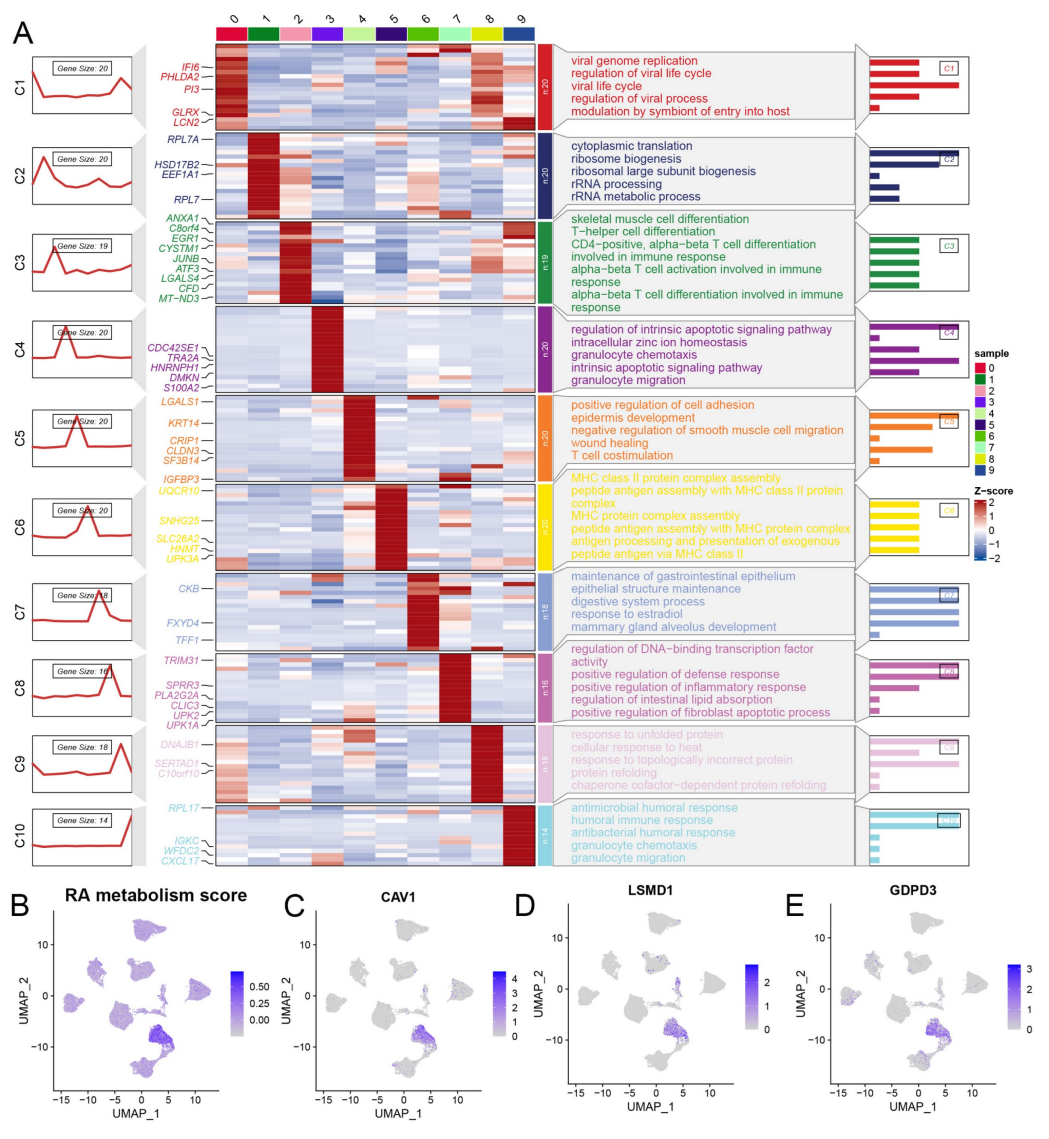
(A) Potential biological function of epithelial cell subsets of bladder tumor. (B-E) RA metabolic fraction and markers of cluster4 in bladder tumor epithelial cell subsets.

We conducted a comprehensive analysis of scRNA-seq data, identifying 21 distinct subclusters (**Figure 2A**), which were classified into five primary cell types: cancer epithelial cells, myeloid/macrophages, fibroblasts, endothelial cells, and T cells (**Figure 2B, 2E**). When focusing on cancer epithelial cells, we further identified 10 subclusters, each associated with different biological functions such as viral life cycle regulation, cytoplasmic translation, and cell differentiation (**Figure 2C, Figure 3A**). Interestingly, clusters 4, 5, and 9 were significantly associated with overall survival in BLCA patients (**Figure 2D**), underscoring the relevance of these subclusters to prognosis. Notably, cluster 4 had a significantly higher RA metabolism score (**Figure 3B-E**), suggesting that increased RA metabolism may be linked to poor prognosis in BLCA patients. This finding adds to the growing body of literature suggesting that RA signaling and metabolism could serve as critical prognostic indicators in cancer, including BLCA.

### Exploring potential therapeutic target associated with RA metabolism

**Figure 4:**
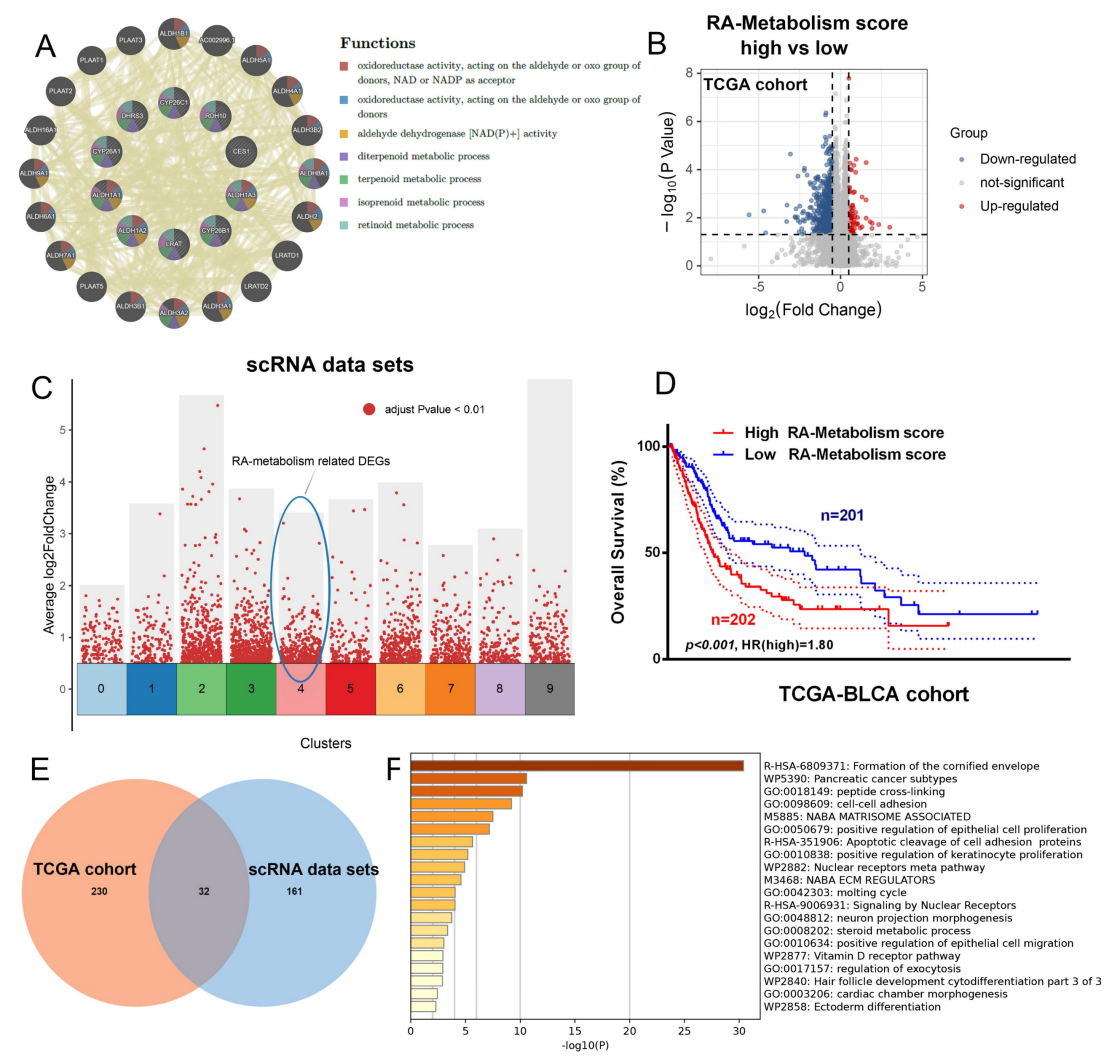
** Exploring potential therapeutic target associated with RA metabolism.** (A) GeneMania analysis of RA metabolism related genes. (B) The DEGs between high and low RA metabolism score group. (C) DGEs in bladder tumor epithelial cell subsets cluster4. (D) Overall survival curves for the high and low RA metabolism score groups (***p***<0.001, HR=1.80, Log-rank tests). (E) Venn diagrams showing the DEGs common in (B) and (C). (F) Metascape function enrichment of the above DEGs.

The potential therapeutic significance of RA metabolism-related genes was explored by conducting GeneMania analysis, which revealed strong interconnectivity among these genes, particularly in the context of oxidoreductase activity (**Figure 4A**). To explore the gene expression changes related to RA metabolism, we investigated the differentially expressed genes associated with it at both the single-cell transcriptome and bulk transcriptome levels (**Figure 4B, C**). This suggests that RA metabolism genes are functionally clustered and may have overlapping roles in cellular redox processes, potentially influencing tumor progression. Further analysis of the TCGA bladder cancer cohort showed that high RA metabolism scores were significantly correlated with worse patient outcomes (**Figure 4D**, p < 0.05, HR = 1.8), indicating that RA metabolism may serve as a prognostic marker. We identified 32 common differentially expressed genes (DEGs) related to RA metabolism, integrating both TCGA and scRNA-seq datasets (**Figures 4E**). These genes were enriched in processes such as the formation of the cornified envelope, which has been linked to cancer cell differentiation and survival. This highlights the potential role of RA metabolism in driving malignant transformation. Furthermore, based on the Metascape functional enrichment analysis results (**Figure 4F**), it suggests that RA metabolism may be involved in processes such as Formation of the cornified envelope, which may promote tumorigenesis.

### Identifying CYP26B1 as a potential therapeutic target

**Figure 5:**
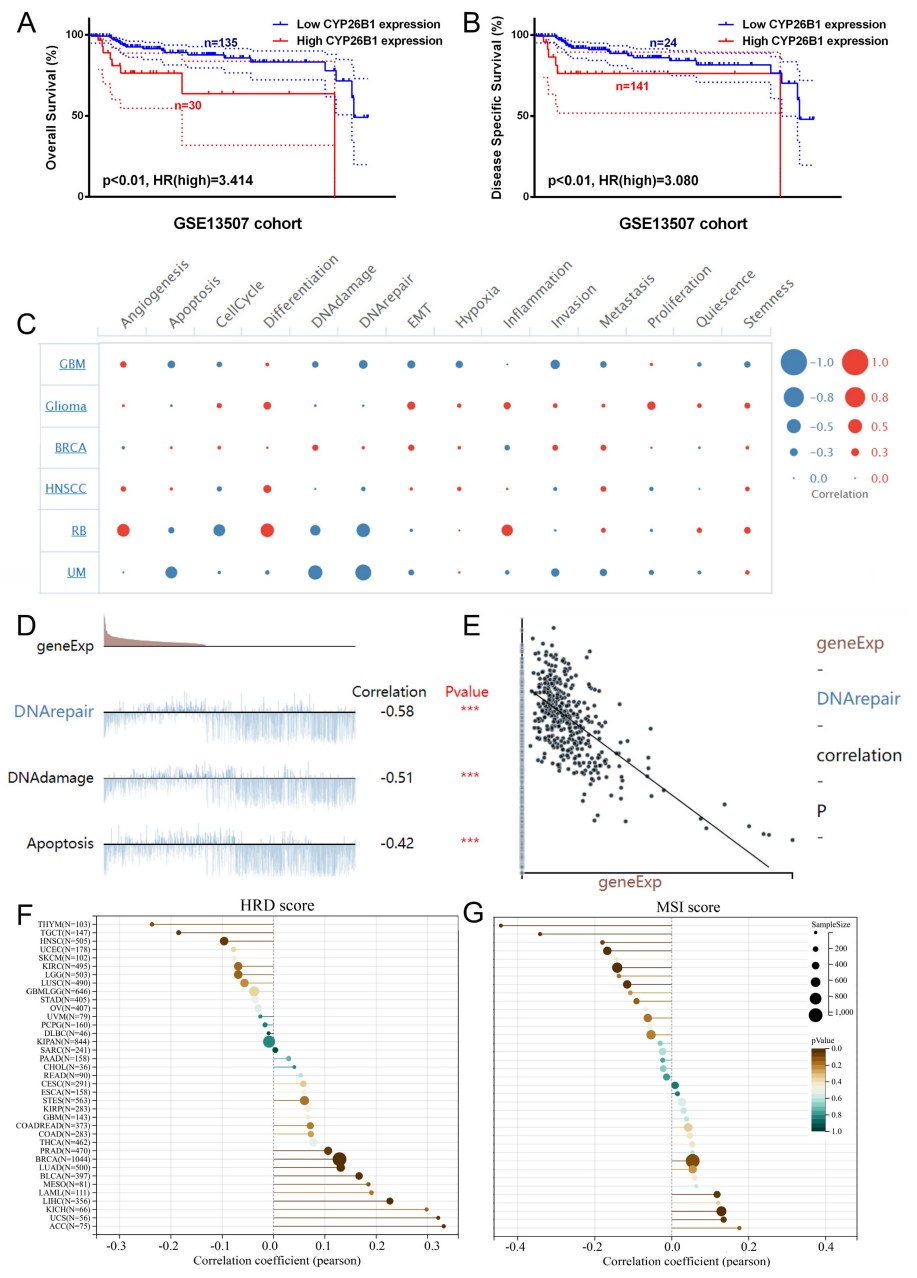
** Identifying *CYP26B1* as a potential therapeutic target.** (A, B) External cohort validation of OS and DSS of CYP26B1 in bladder cancer, respectively (Log-rank tests). (C) Single-cell transcriptional data to assess the potential function of CYP26B1. (D and E) The correlation between expression level of CYP26B1 and DNA repair (Spearman correlation test). (F) The correlation between CYP26B1 and HRD score (Spearman correlation test). (G) the correlation between *CYP26B1* and MSI score (Spearman correlation test).

Among the 32 DEGs, CYP26B1, a key enzyme involved in RA metabolism, emerged as a potential therapeutic target for BLCA. External cohort validation revealed that high expression of CYP26B1 was significantly associated with poorer overall survival (OS) and disease-specific survival (DSS) (**Figure 5A, B**). These findings support previous studies that have linked CYP26B1 expression to poor prognosis in various cancers. Single-cell analysis further revealed that CYP26B1 expression was negatively correlated with DNA damage repair (**Figure 5C-E**), suggesting that CYP26B1 may contribute to tumor progression by impairing the DNA damage response. This is particularly relevant given the pivotal role of DNA repair mechanisms in cancer. Moreover, CYP26B1 expression was inversely correlated with HRD (homologous recombination deficiency) scores in BLCA and BRCA (**Figure 5F**), while in other cancer types like BRCA and colon adenocarcinoma, CYP26B1 expression was positively correlated with MSI (microsatellite instability) scores (**Figure 5G**). These observations suggest that CYP26B1 may contribute to genomic instability, a hallmark of cancer.

### CYP26B1 promotes BLCA tumor stemness

**Figure 6:**
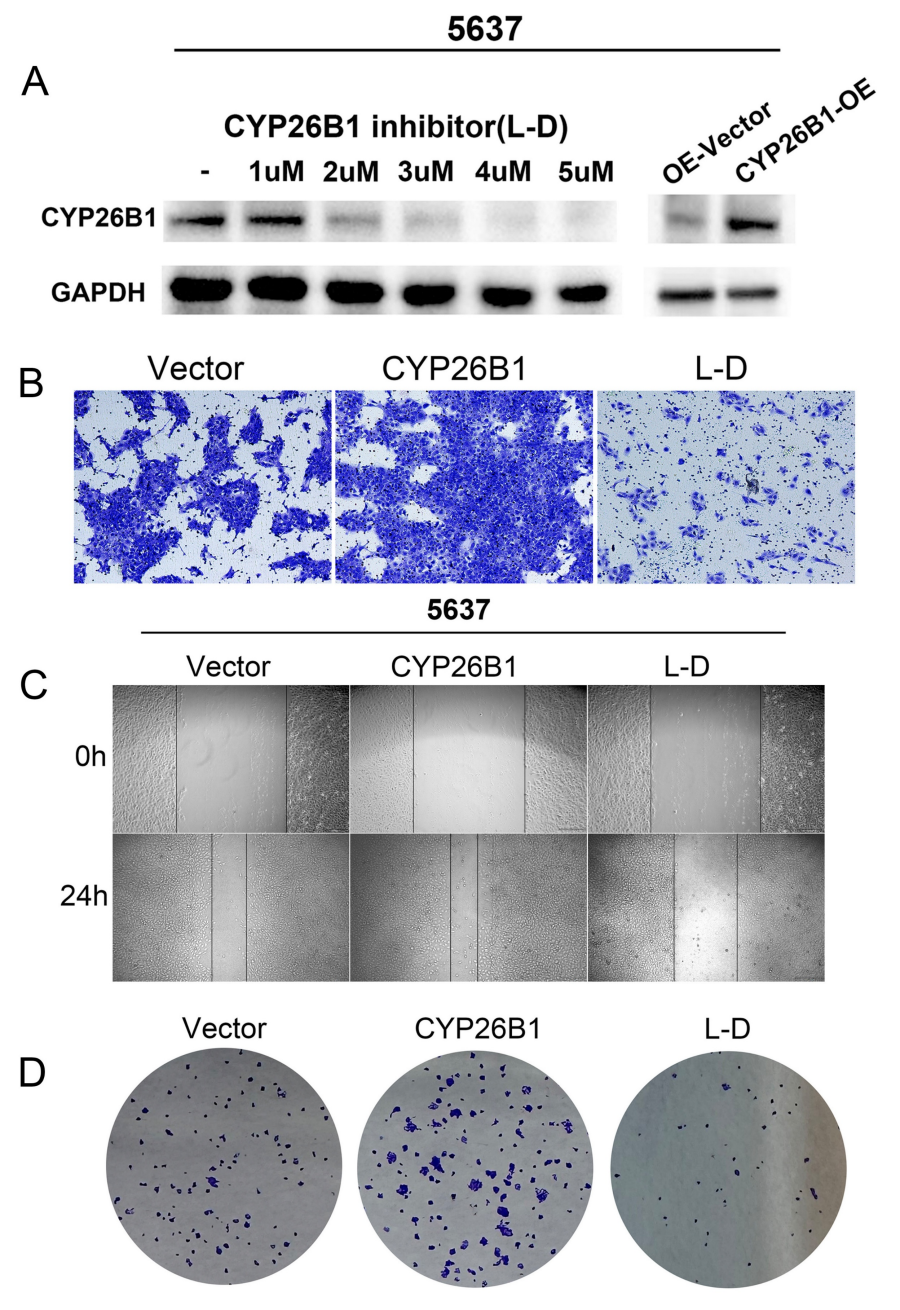
** CYP26B1 promote BLCA migration and proliferation in 5637 cells.** (A) Validation of inhibition and overexpression efficiency by western blot. (B) Transwell assay to explore cell migration ability in 5637 cell after overexpression and inhibition of CYP26B1. (C) Wound-healing assay to explore cell migration ability in 5637 cell after overexpression and inhibition of CYP26B1. (D) Colony formation assay to explore proliferation ability in 5637 cell after overexpression and inhibition of CYP26B1.

**Figure 7:**
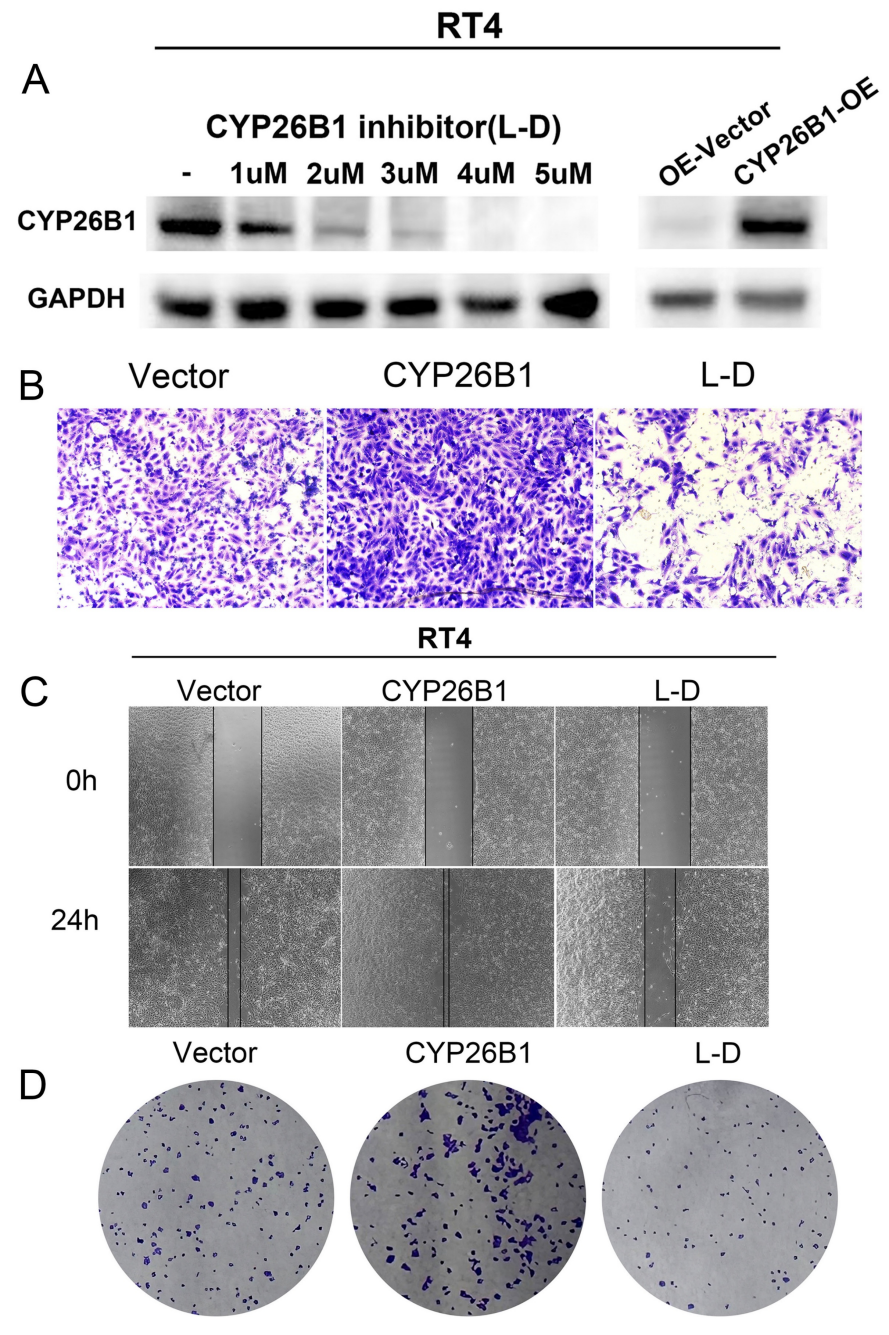
** CYP26B1 promote BLCA migration and proliferation in RT4 cells.** (A) Validation of inhibition and overexpression efficiency by western blot. (B) Transwell assay to explore cell migration ability in RT4 cell after overexpression and inhibition of CYP26B1. (C) Wound-healing assay to explore cell migration ability in RT4 cell after overexpression and inhibition of CYP26B1. (D) Colony formation assay to explore proliferation ability in RT4 cell after overexpression and inhibition of CYP26B1.

*In vitro* experiments were undertaken to explore the potential role of CYP26B1 in bladder cancer (BLCA), utilizing MIBC (5637) and NMIBC (RT4) cell lines. Western blot analysis demonstrated a significant reduction in CYP26B1 expression upon treatment with the CYP26B1 inhibitor (L-D) in both 5637 and RT4 cells, as depicted in **Figures 6A and 7A**, respectively. Utilizing transwell and wound healing assays, it was observed that upregulation of CYP26B1 facilitated the migration of both 5637 and RT4 cells, whereas L-D treatment hindered the migration of both cell types (**Figure 6B, C; Figure 7B, C**).

The CCK-8 assay confirmed that CYP26B1 overexpression can enhance the proliferation ability of bladder cancer cells (**Supplementary figure 3A and C**). Additionally, colony formation assays revealed that CYP26B1 overexpression boosted the proliferation of 5637 and RT4 cells, whereas L-D treatment curbed the proliferation of 5637 and RT4 cells, as illustrated in **Figure 6D and 7D (The statistical charts are in Supplementary figure 3B and D)**. These findings collectively support the notion that CYP26B1 promotes the tumor stemness of BLCA. To further strengthen these conclusions, additional *in vivo* data were included in the revised manuscript. Specifically, we utilized a subcutaneous xenograft model in nude mice to investigate the effect of CYP26B1 on bladder cancer growth. The results confirmed that overexpression of CYP26B1 promoted bladder tumor growth (**Supplementary figure 2**). Moreover, treatment with Liarozole dihydrochloride (40 mg/kg, p.o.) significantly inhibited the growth of bladder tumors in the nude mice, further supporting our *in vitro* findings.

### Exploring the association between CYP26B1 and the tumor microenvironment

**Figure 8:**
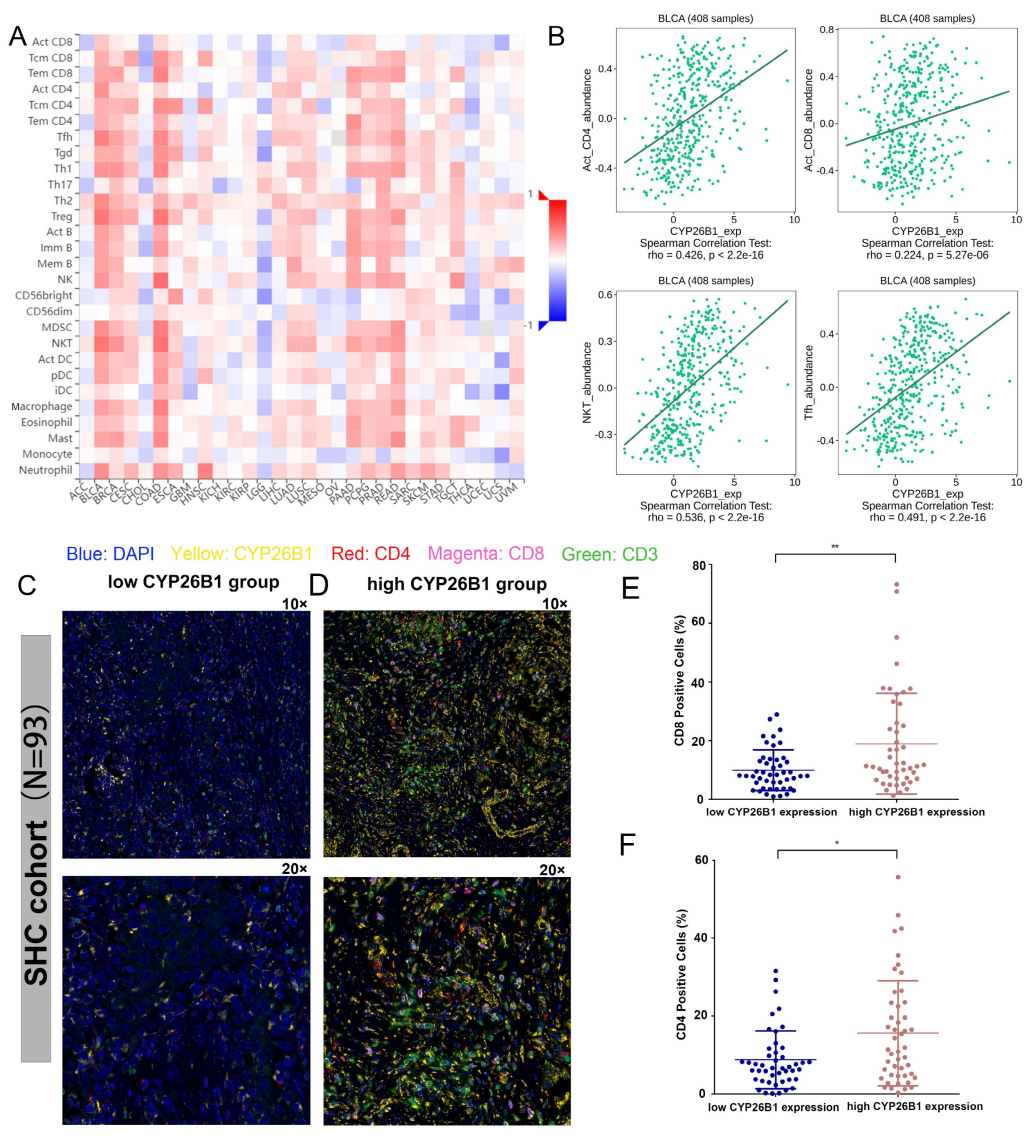
** Exploring the association between CYP26B1 and the tumor microenvironment.** (A) The association between the expression level of CYP26B1 and various types of immune cell infiltration in most tumors. (B) The association between CYP26B1 expression and CD4+T cells, activated CD8+T cells, NKT cells, and Tfh cells in bladder cancer. (C, D) Multiplex immunohistochemical images indicating the associations between CYP26B1 and tumor infiltrating immune cells including CD4+ and CD8+ T cells. (E, F) Quantification of mIHC signals for estimating the associations between CYP26B1 and tumor infiltrating immune cells including CD4+ and CD8+ T cells.

Recent advances in immunotherapy have underscored the importance of understanding the tumor microenvironment (TME) in cancer therapy. We found that CYP26B1 expression was positively correlated with immune cell infiltration, particularly CD4+T cells, CD8+T cells, NKT cells, and Tfh cells in BLCA (**Figure 8A, B**). This suggests that CYP26B1 may play a role in modulating the immune response within the TME, potentially enhancing the effectiveness of immune checkpoint inhibitors.

Additionally, immunohistochemical analysis of samples from our cohort confirmed a positive correlation between CYP26B1 expression and infiltration of CD8+T cells and CD4+T cells (**Figure 8C-F**), further supporting its role in shaping the immune microenvironment. Interestingly, high CYP26B1 expression was associated with improved prognosis in BLCA patients treated with immune checkpoint inhibitors (**Supplementary figure 3E**), highlighting its potential as a biomarker for immunotherapy response.

## Discussion

Retinoic acid is widely recognized as a dietary regulator that contributes to various signaling mechanisms to maintain intracellular biological needs. RAS crosses the nuclear membrane through RARs, binds to specific sequences, and participates in gene regulation. Some studies have shown that genes involved in cell regulation, proliferation, repair, and maintenance have conserved rare domains in their promoter regions, thus activating gene transcription 29. This leads to the transcriptional activation of several genes involved in proliferation mechanisms 30. Additionally, some studies have shown that this mechanism is involved in the acquisition of drug resistance in osteosarcoma, a type of cancer 31, 32. In this study, we have explored the relationship between RA metabolism and bladder cancer for the first time. At the genomic level, we found that the mutation rate of genes related to RA metabolism is nearly 20% in bladder cancer, indicating that the relationship between RA metabolism-related genes and the occurrence of bladder cancer is closely linked. At the transcriptomic level, we found that RA metabolism is significantly correlated with higher tumor stage, suggesting a potential relationship between RA metabolism and the malignant phenotype of tumors. Overall, the analysis results suggest that RA metabolism may provide a new pathway for developing therapeutic targets for bladder cancer.

Cytochrome P450 family 26 (CYP26) is a member of the cytochrome P450 protein superfamily, which functions as a hydroxylase and inactivates all-trans retinoic acid, thus regulating the concentration of RA 33. There are three members of the CYP26 family in mammals: CYP26A1, CYP26B1, and CYP26C1 34. Among them, CYP26B1 is a risk factor for esophageal squamous cell carcinoma and increases the risk of oral squamous cell carcinoma 35, 36, but its association with BLCA was unclear. Retinoic acid has an important role in DNA damage repair and effectively protects cells from mutation or carcinogenesis. When DNA damage cannot be effectively repaired, cells may contribute to tumorigenesis. The normal metabolism and function of retinoic acid are crucial for maintaining the integrity of DNA, and the abnormality of retinoic acid metabolism may hinder DNA damage repair and increase the risk of tumor. In this study, we found that CYP26B1 is significantly associated with the poor prognosis of bladder cancer and have used cell phenotypic experiments to confirm that over-expression of CYP26B1 can promote the proliferation and migration abilities of tumor cells. This is supported by the significant inhibition of bladder tumor cell growth when CYP26B1 expression is inhibited. Therefore, CYP26B1 can be considered a potential therapeutic target for bladder cancer, similar to its discovery in other malignancies 37. We provide compelling evidence that CYP26B1 is a key player in bladder cancer progression, offering a novel perspective on RA metabolism in this malignancy.

Retinoic acid metabolism not only influences the development and progression of tumors but also affects the composition of the tumor microenvironment, which is crucial for the application of immune checkpoints. It was reported that all-trans retinoic acid therapy inhibited tumor growth in syngeneic immunoactive mice but did not inhibit tumor growth in immunodeficient mice 38. Tumor microenvironment suggests that the loss of CD8+ T cells can antagonize the anti-tumor effect of all-trans retinoic acid on syngeneic mice 39, 40. This suggests that all-trans retinoic acid may be closely related to the anti-tumor effect of immune cells. In this study, based on the TCGA cohort and external validation cohorts, we found a significant positive correlation between CYP26B1 expression and the infiltration of immune cells within tumors. Furthermore, CYP26B1 is able to indicate better prognosis in patients undergoing immunotherapy, indicating that CYP26B1 may facilitate the infiltration of immune cells and thus enhance the effectiveness of immune checkpoint inhibitors.

This study provides novel insights into the role of CYP26B1 in bladder cancer, particularly its impact on both tumor progression and immune cell infiltration, which has not been thoroughly explored in this context. By linking RA metabolism to immune response modulation, we propose a new avenue for therapeutic intervention targeting CYP26B1. However, several limitations should be acknowledged. Our findings are primarily based on bioinformatic analyses and cell phenotypic experiments, and further *in vivo* studies are necessary to validate these results. Additionally, the mechanisms through which CYP26B1 influences immune cell dynamics require more detailed exploration. Future research should focus on the potential for RA-based therapies in combination with immune checkpoint inhibitors and explore the role of CYP26B1 in various stages of bladder cancer development. This could pave the way for novel therapeutic strategies aimed at improving the efficacy of current immunotherapies and addressing RA metabolism dysregulation in bladder cancer.

## Conclusions

In this study, we conducted the first exploration of the potential role of RA metabolism in bladder cancer. We explored the landscape of genes related to RA metabolism in bladder cancer, and discovered that RA metabolism is closely related to the stemness of bladder cancer cells. The key enzyme of RA metabolism, CYP26B1, can be used as a potential therapeutic target for bladder cancer, and its expression can effectively promote the stemness of bladder cancer. Furthermore, we found that there is a significant positive correlation between CYP26B1 and an increase in tumor-infiltrating lymphocytes. Additionally, CYP26B1 can indicate better prognosis for immunotherapy patients. Overall, we have explored the landscape of genes related to RA metabolism in bladder cancer for the first time and discovered that RA metabolism pathway could be used as a potential therapeutic target for bladder cancer.

## Supplementary Material

Supplementary figures and table.

## Figures and Tables

**Table 1:** Data collection.

Data Set ID	Type of Data	Data Base	Number of BLCA samples
TCGA-BLCA	RNA-seq	TCGA	403
IMvigor210	RNA-seq	IMvigor210	292
GSE13507	Expression profiling by array	GEO	165
GSE130001	scRNA-seq (10x Genomics)	GEO	2
GSE135337	scRNA-seq (10x Genomics)	GEO	8

## Abbreviations

Bladder cancer is referred to as BLCA: differentially expressed genes are known as DEGs, disease-specific survival is abbreviated as DSS, muscle-invasive bladder cancer is denoted as MIBC, Liarozole dihydrochloride is represented as L-D, overall survival is called OS, progression-free survival is referred to as PFS, protein protein interaction is known as PPI, Retinoic acid is abbreviated as RA, the cancer genome atlas is referred to as TCGA, and tumor microenvironment is denoted as TME
