# Supplementary material for: Retinoic acid metabolism related gene CYP26B1 promotes tumor stemness and tumor microenvironment remodeling in bladder cancer
Source: J Cancer. 2025 Apr 22;16(8):2476–91. doi: 10.7150/jca.101406 (PMC12170498; doi:10.7150/jca.101406)
Supplement: Supplementary file 1 — Supplementary figures and table. [file jcav16p2476s1.pdf]

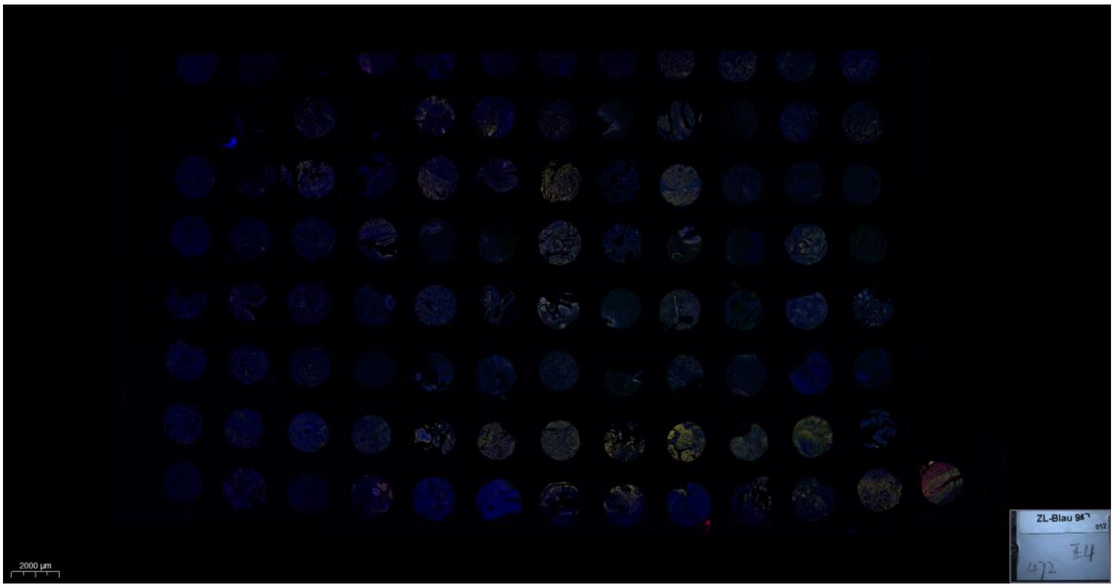

Supplementary figure 1. The scanning image of mIHC.

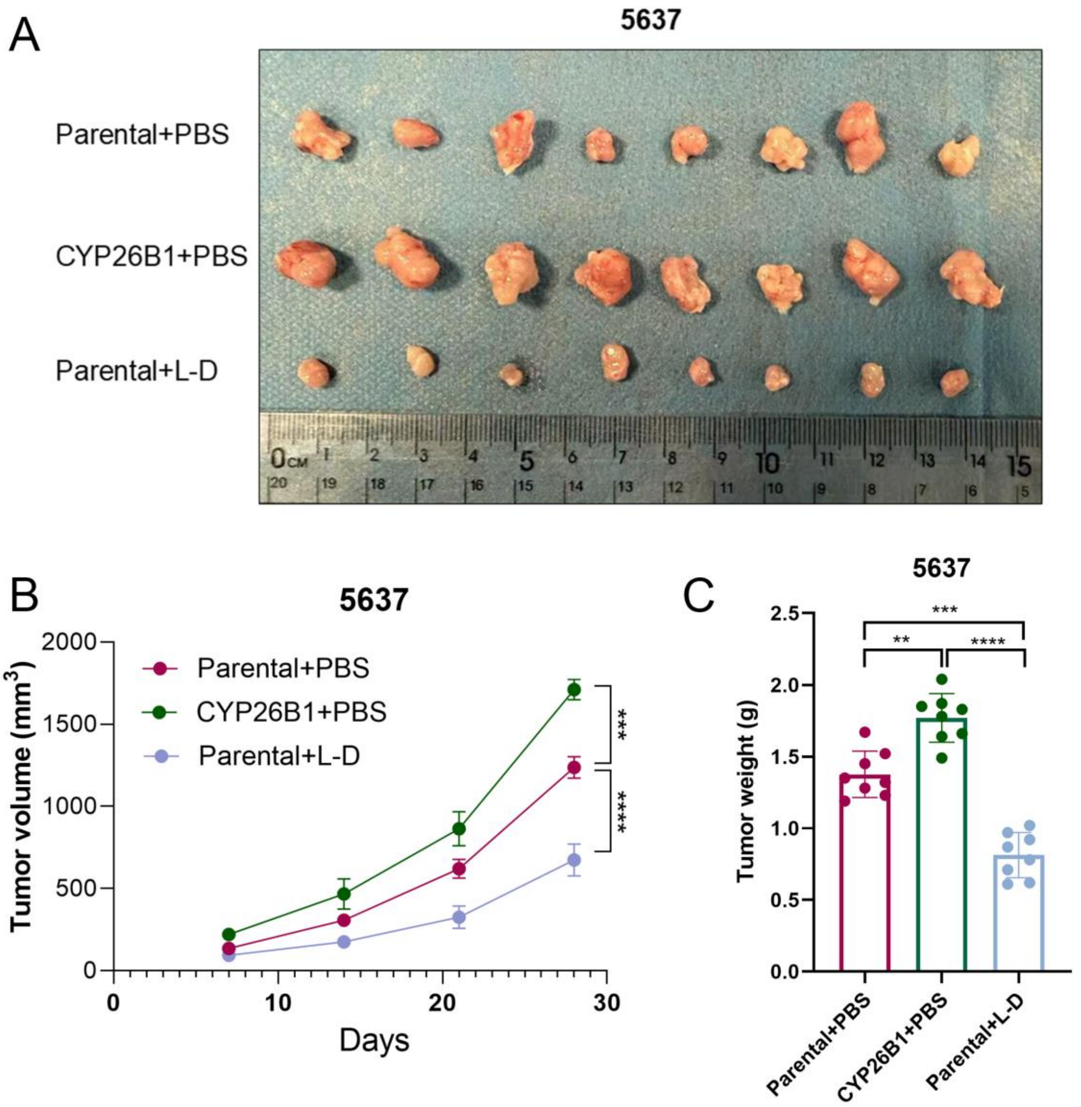

Supplementary figure 2. In vitro experiments confirmed that CYP26B1 promoted bladder tumor growth. A, Tumor formation photography; **B**, tumor growth curve; **C**, tumor weight bar chart.

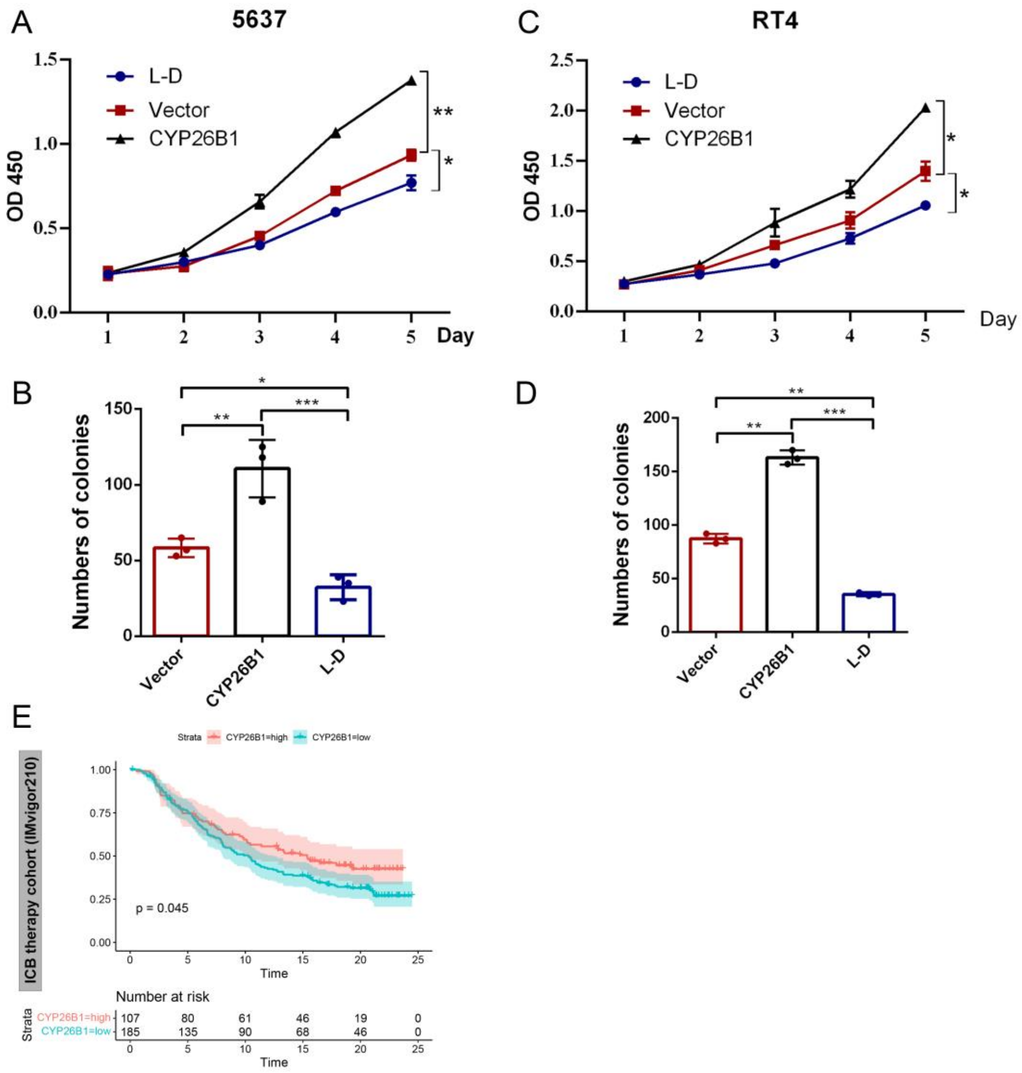

Supplementary figure 3. In vitro experiments confirm the effect of CYP26B1 on tumor proliferation. A-B, CCK-8 and colony formation assays confirm the effect of CYP26B1 on 5637 tumor proliferation. **C-D**, CCK-8 and colony formation assays confirm the effect of CYP26B1 on RT4 tumor proliferation. **E**, The relationship between CYP26B1 expression and prognosis in ICB-treated populations.

TMA Row	TMA Column	Left (pixels)	Top (pixels)	Total Cells	CD4+ Cells	CYP26B1+ Cells	CD8+ Cells	CD3+ Cells	% CD4 Positive Cells	% CYP26B1 Positive	% CD8 Positive	% CD3 Posit
7	3	23281	65147	10330	491	891	102	793	4.753146	8.628737	0.987415	7.674734
7	7	56724	66041	14541	945	1333	1383	2581	6.498865	9.166767	9.511038	17.749261
6	7	56886	57719	7044	100	846	211	594	1.419648	12.016769	2.989779	8.432709
2	8	63146	24682	8736	81	1098	508	666	0.927198	12.570146	5.817307	7.621337
8	7	56671	74069	8951	279	1209	601	1041	3.11697	13.507677	6.712099	11.630879
7	4	31386	65696	11950	820	1671	944	2024	6.861925	13.986486	7.899582	16.937239
5	5	40002	50037	11453	375	1831	203	804	3.274251	15.988126	1.772461	7.018947
3	7	56635	33048	13674	809	2220	2936	3134	5.914875	16.232906	21.471405	22.916046
9	3	22589	81200	19805	1577	3335	1427	3828	7.962636	16.840391	7.205251	19.327947
5	7	56873	49591	7874	639	1506	1108	2073	8.115316	19.12785	14.069088	26.324613
3	5	40115	33833	16370	40	3622	281	575	0.244349	22.125713	1.716555	3.512523
9	7	55988	82157	8791	823	1967	2402	2824	9.361847	22.377716	27.323399	32.128199
5	2	15410	50575	10087	805	2312	840	2261	7.980569	22.915812	8.32755	22.417171
4	7	56995	41230	13540	985	3138	506	1650	7.273262	23.175005	3.734119	12.186115
9	5	38894	81920	15540	2023	4019	574	3048	13.018018	25.860521	3.693694	19.611326
5	1	7407	50496	9898	1641	3005	705	2614	16.579107	30.359869	7.122651	26.406547
2	5	39994	24959	7842	928	2504	1018	2409	11.833715	31.925003	12.981382	30.72048
10	7	55907	90301	14660	532	4941	505	1519	3.628922	33.704884	3.447475	10.358799
11	3	22377	97551	10821	883	3750	1992	2324	8.163017	34.652637	18.408649	21.474725
2	1	7886	26434	14834	866	5686	558	1391	5.840367	38.328156	3.761629	9.379668
2	2	15913	26378	9279	272	3793	852	1167	2.932428	40.882294	9.182024	12.576786
6	4	31195	58072	8309	519	3568	691	985	6.246239	42.94701	8.316283	11.854615
13	7	54249	114465	11195	672	4885	1584	1908	6.00268	43.637154	14.149174	17.045109
5	3	23877	49823	19558	728	8752	2621	4622	3.721241	44.748005	13.40321	23.633297
3	4	31563	34157	16943	4960	7619	1356	4583	29.274626	44.969513	8.003305	27.051998
7	2	15368	66187	6461	488	2909	374	886	7.55301	45.022861	5.788578	13.707785
6	2	15479	58492	5833	567	2714	1382	2055	9.729126	46.524738	23.692783	35.224926
5	4	31383	49740	6173	1347	2922	491	1879	21.820833	47.340427	7.953993	30.438199
7	5	39455	65772	9681	571	4663	2794	3316	5.896912	48.161381	28.861069	34.249046
9	2	14542	81814	9507	1949	4597	1304	2861	20.500683	48.357441	13.716209	30.091722
5	6	48320	49815	6064	321	2960	166	577	5.293536	48.813301	2.737467	9.514511
4	1	7886	42514	10460	238	5159	820	1178	2.275335	49.317249	7.839388	11.260038
3	2	15589	34369	17761	40	8836	196	325	0.225213	49.747742	1.103541	1.829852
3	8	65094	32922	11496	425	5749	1402	1540	3.696938	50.009163	12.195546	13.395964
12	8	62541	106911	10658	103	5423	324	537	0.96641	50.886551	3.040344	5.03753
7	6	48056	66103	10390	14	5295	960	1444	0.134747	50.957855	9.239654	13.896053
13	6	45921	114596	12109	703	6310	1383	2466	5.805599	52.107414	11.421257	20.362623
11	7	55217	98587	16776	2884	8770	3614	6916	17.191225	52.275055	21.542681	41.227468
8	5	39019	73330	7070	2232	3722	645	2878	31.570015	52.650646	9.123055	40.702971
11	2	14847	97240	6990	531	3937	565	1426	7.596567	56.320621	8.082975	20.39485
10	4	30552	89267	11306	2967	6416	2183	4962	26.242702	56.745068	19.308332	43.890147
12	7	54850	106802	13707	1169	7996	781	1908	8.528489	58.333332	5.697819	13.918217
8	3	22151	73569	10065	1616	5896	1277	2278	16.050671	58.580498	12.687531	22.632887
2	7	55214	24775	7532	495	4498	808	1401	6.571959	59.72464	10.727562	18.602762
11	4	30241	97579	9088	957	5573	571	1747	10.53037	61.318584	6.28301	19.224032
8	8	64370	73980	24876	2899	15568	867	196	11.65525	62.581997	3.485287	0.787908
13	5	38179	113995	6554	81	4149	1275	1143	1.235886	63.304852	19.449497	17.434239
7	8	64881	65655	10976	1246	6967	2855	3671	11.352041	63.470814	26.011297	33.443513
6	8	65209	57719	11300	832	7268	1909	3104	7.362832	64.315582	16.893805	27.469027
13	8	61657	114621	6319	178	4092	2383	2235	2.822915	64.763672	37.706916	35.363982
11	6	46974	98466	8665	235	5685	113	777	2.71206	65.61351	1.304097	8.961916
4	8	64416	40678	9504	885	6278	1048	2272	9.311869	66.061134	11.026936	23.903199
8	2	14772	73911	16879	1189	11697	2084	3685	7.044256	69.299484	12.346703	21.830974
6	6	48879	58056	2832	665	1965	284	1021	23.481638	69.399139	10.028249	36.052261
11	5	38447	98265	8281	2658	5796	761	3112	32.097572	69.985855	9.189712	37.576742
4	6	48281	41650	6835	1123	4831	363	859	16.43014	70.684311	5.3109	12.567666
12	6	46175	106276	11507	3043	8191	1190	3897	26.444773	71.178459	10.341531	33.864778
8	1	7191	73291	5481	225	3984	429	539	4.10509	72.694901	7.827039	9.842729
9	6	47619	82117	6373	1488	4642	1596	2772	23.348501	72.837303	25.04315	43.490193
6	1	7789	57924	5457	937	4052	398	1301	17.170607	74.251915	7.293385	23.848268
6	5	39861	57891	7532	2500	5647	1077	3264	33.191715	74.967628	14.298991	43.335102
4	4	31712	41761	4394	859	3306	219	673	19.549385	75.239006	4.984069	15.306782
7	1	7309	65744	9143	1672	6887	1545	2805	18.287214	75.329819	16.898174	30.681067
11	8	63264	98671	7397	656	5600	3416	3240	8.868461	75.712631	46.17818	43.798432
6	3	23329	57825	6717	172	5099	2233	1966	2.560667	75.911583	33.237968	29.265148
2	3	23988	26602	3948	325	3005	2176	1535	8.232017	76.105621	55.116515	38.879433
10	8	63748	90479	11884	2670	9154	366	3133	22.467182	77.02726	3.081733	26.361495
2	4	32127	26431	2843	291	2252	267	813	10.249737	79.198265	9.391488	28.596554
3	3	23962	34043	7216	450	5722	1655	1501	6.236142	79.29528	22.937916	20.802661
12	5	38512	106151	5893	2505	4709	623	2315	42.50806	79.90081	10.571865	39.281012
8	4	31426	73821	4597	1202	3690	517	1162	26.138786	80.267975	11.246465	25.277355
3	1	7886	34514	8266	1183	6647	484	1727	14.311638	80.418823	5.855311	20.892815
1	8	63092	16190	10178	4671	8233	7445	5162	45.895264	80.892334	73.151894	50.7192
13	1	7191	112386	13841	41	11223	1317	979	0.296221	81.084	9.515208	7.072249
10	5	38703	89772	8363	2974	6825	981	2577	35.561401	81.609833	11.73024	30.815258
10	3	22523	89152	5652	222	4724	2129	1911	3.927813	83.581177	37.66242	33.813164
4	3	23921	42259	7787	3255	6523	945	1847	41.800438	83.766899	12.135611	23.719019
9	4	30661	81479	4585	2551	3858	3245	2413	55.637951	84.138206	70.764449	52.631405
12	1	7031	104201	3341	155	2898	1266	1451	4.639329	86.73745	37.897935	43.419036
8	6	47960	74039	10274	1109	9055	677	1897	10.794238	88.131462	6.589449	18.464085
12	3	22058	104968	2435	476	2225	115	711	19.548254	91.36512	4.722793	29.188912
9	1	7191	81688	7503	1235	6902	531	1909	16.460083	91.991806	7.077169	25.438225
10	1	6871	89002	8750	2725	8060	3194	4121	31.142857	92.119728	36.502857	47.097143
13	3	22058	112871	8072	1303	7519	565	1920	16.14222	93.154686	6.999505	23.787413
10	6	47158	90162	3070	157	2871	735	854	5.114007	93.526886	23.944625	27.824104
3	6	48089	32824	15869	224	15098	5680	6203	1.411557	95.144066	35.794001	39.087781
4	2	15956	42646	2358	363	2270	55	544	15.394403	96.271721	2.332485	23.070398
2	6	47533	24867	4440	201	4286	416	339	4.527027	96.527199	9.36937	7.635135
9	8	64179	82057	20840	2475	20181	2362	5432	11.8762	96.838341	11.333973	26.065259
13	4	29783	113592	11583	187	11295	3764	4344	1.614435	97.516029	32.4921	37.500734
5	8	64780	49463	20513	988	20073	718	1527	4.816458	97.856331	3.500219	7.445961
4	5	39878	42167	8269	541	8103	436	813	6.542508	97.991882	5.272705	9.829725
12	4	30071	105877	7927	130	7811	1408	1880	1.639965	98.538239	17.766116	23.719061

Supplementary table 1. mIHC staining fluorescence signal statistics table.
